# Consumer Purchasing Behavior Toward Green Environment in the Healthcare Industry: Mediating Role of Entrepreneurial Innovation and Moderating Effect of Absorptive Capacity

**DOI:** 10.3389/fpubh.2021.823307

**Published:** 2022-02-03

**Authors:** Muddassar Sarfraz, Mohsin Raza, Rimsha Khalid, Tong Liu, Zeyu Li, Lubna Niyomdecha

**Affiliations:** ^1^College of International Students, Wuxi University, Wuxi, China; ^2^Faculty of Management Sciences, Phuket Rajabhat University, Phuket, Thailand; ^3^Department of Business Management, Limkokwing University of Creative Technology, Cyberjaya, Malaysia; ^4^Office of Academic Affairs, Zhenjiang College, Zhenjiang, China; ^5^Jingjiang College, Jiangsu University, Zhenjiang, China; ^6^Department of Human Resource Management, Faculty of Management, Phuket Rajabhat University, Phuket, Thailand

**Keywords:** consumer purchasing behavior, pharmaceutical, healthcare, green environment, sustainable economy, entrepreneurial innovation

## Abstract

Entrepreneurial innovations lead to transformations in the existing business model, involving them integrating the new knowledge into the established entrepreneurial activities. The current study combines marketing strategies, entrepreneurial innovation, absorptive capacity, and consumer purchasing behavior in the health sector. This study investigates the impact of packaging and labeling strategies on entrepreneurial innovation and consumer purchasing behavior. Therefore, this study also investigates the mediating effect of entrepreneurial innovation between packaging and labeling strategies and consumer purchasing behavior. The study examines the moderating role of absorptive capacity between entrepreneurial innovation and consumer purchasing behavior. While using convenience sampling, this study used the consumer of medications as a sample from China. The research adopted a quantitative method to conduct the study analysis. A well-structured questionnaire with a 5-point Likert scale has used for the study analysis. The adopted questionnaires were utilized in data collection with 702 with a 70% response rate, and Smart PLS was used to analyze the data. The findings of this study indicate that packaging and labeling strategies significantly influence entrepreneurial innovation and consumer purchasing behavior. Entrepreneurial innovation significantly mediates the packaging and labeling strategies and consumer purchasing behavior. The moderating role of absorptive capacity significantly affects entrepreneurial innovation and consumer purchasing behavior. The implications of the investigated model with innovative marketing strategies give new insights for managerial, policymakers, and research perspectives.

## Introduction

Due to the progressing globalization and increasing competition nowadays, the need for effective product recognition has become essential, with product packaging playing a critical role in driving the vehicle of manufacturing businesses. All the processed commodities require formalized packaging from the first phase of product generation to the final distribution. Packaging is an integral component of marketing strategy. It encourages the companies to develop unique products, thereby receiving a favorable buyer response. Consumer purchase behavior is a process of assessing, selecting, and obtaining the product that fulfills the requirement of the buyers. Consumers judge the products based on their needs. The wide extend of products characteristics accessible to consumers respects quality, design, label, packaging, and functionality, thus satisfying the consumers' requirement. Above all, packaging constitutes an instrumental role in communicating the brand attributes to the potential users. Product characteristics capture the consumer's attention in the shape of color, design, packaging, and labeling. Packaging provides a valuable experience to all the buyers, developing a positive consumer perception, thereby pulling the potential consumers toward the brand (1). Notably, producers and manufacturers communicate with the consumers through information sources (i.e., product characteristics). The information provided in the shape of packaging assists the consumer in making the most favorable buying choices. However, packaging strategies at various levels within industries (e.g., pharmaceutical) record positive consumer behavior (2).

Over the years, pharmaceutical products have received significant importance from buyers. The contributing factors (e.g., packaging and labeling) lighten the advancement of industrial production while extending the accessibility of drug items (3). Considerably, the changing dynamics of the global market in the pharmaceutical industry have added complexity in meeting the universal standards of drug production in numerous nations (4). As a result, companies are thoroughly screening the manufacturing processes, fulfilling the safety and quality measures of the stakeholders, thereby differentiating the pharmaceutical brands (e.g., local and global). In pharmaceutical parlance, product packaging (e.g., bottle, vessels, caps) has gained much attention in the production process, making the drugs products differentiate from the competitors. In the healthcare sector, the product packaging reflects the quality of the product. The marketers use different product packaging to gain consumers' attention. Today, based on the product packaging, pharmaceutical products are assumed safe and secure by the consumers. In the pharmaceutical industry, the packaging makes consumers think that the product has a positive effect on consumers' health. In the illustration, the study shows that this sense of healthiness makes the consumer buy the product (5). In particular, product packaging prompts various cues, influencing consumer buying choices (6).

Furthermore, in the health care industry, labeling being a crucial component of packaging eliminates the uncertainty of the end-users while reinforcing positive buying choices. Product label provides meaningful information about the product attributes (e.g., ingredients, taste, quality, date of manufacturing, expiry date) (7). Product labeling is the prime source of providing information to the end-user at the point of purchase (8). In the health care industry, consumers make purchases based on product information. Many individuals buy products that ensure positive consumer health. In particular, the packaging cues provide essential information to the consumer, thus encouraging them to purchase the product. The influence of labeling strategy on consumer health brings positive results. The product's physical appearance and the health claims made on the packaging are perceived to be credible and healthy. Given the articulation, the research shows that the nutrition information provided on the product label records a positive consumer attitude in the shape of a favorable response (9). Perhaps, the crucial information motivates the buyer to make a purchasing decision. Therefore, the labeling strategy influences the consumer's purchasing choice while enabling the buyers to pay for labeled products (10).

Additionally, research shows that packaging with innovation has created a new dimension in which entrepreneurial innovation has added tremendous value within the supply chain (11). Entrepreneurship aims at benefiting from the opportunities, while product innovation refers to the value-added activities, subsequently differentiating one product from the other product. Products without innovation tend to be left behind by the consumer. Consistently, the packaging innovation in the pharmaceutical market ensures a safe and premium product performance. To improve the product packaging, entrepreneurs encourage the marketers to incorporate consumers' needs by ensuring consumers' safety. Given the statement, the study shows that efficient and intelligent pharmaceutical packaging in the healthcare service motivates consumers to buy safe, secure, and healthy products (12). Indeed, entrepreneurial innovation activities develop modifications, creating favorable product variations (e.g., attractive packages), thus extending buyers' interest in product purchases (13, 14).

However, consumers' favorable decisions are not only influenced by innovation, despite being persuaded by the positive information available to them at the point of purchase. Product developers drive consumer interest by maintaining information transparency. Increasing consumer awareness of product information supports the companies in sharing transparency-based procedures (e.g., certifications) among supply chain actors (15, 16).

In particular, for firms to innovative absorptive capacity forms an integral component in implementation organizations' learning capabilities into new business procedures. It enables the manufactures to develop a solid knowledge foundation (17) while rigorously integrating acquired knowledge into product innovation. The firms' capacity to acquire and disperse new knowledge and learning capabilities compels the businesses to innovate, thus strengthening the effect of entrepreneurial innovation on consumer behavior (18–20).

However, various analysts have investigated the proposed research topic (i.e., packaging). Every research has illustrated a specific conclusion, but few crevices had recognized in the previous writings. These gaps explain that prior research has been limited to product features only, such as colors and graphics (21). Despite the increasing importance of this subject in marketing, the inconsiderable interest of scholars from the management point of view had recorded (22). Unfortunately, this limited research dealing with consumer behavior in the pharmaceutical industry needs to address. As a result, consumer behavior regarding product characteristics needs to be acknowledged. From a strategic point of view, comprehensive research needs to address the issue regarding the relationship between firms' packaging strategy and its effect on consumer behavior (23, 24).

In recent years, eco-friendly packaging has gained immense significance in the world's healthcare sector. A green environment in the healthcare industry enhances the consumers' consumption patterns, thereby fostering the demand for protecting the environment. The green environmental tools used by the marketers ensure the consumer's psychological well-being, thus leading them to make a favorable purchase decision. However, research shows that the consumer demand for a healthy environment has not been satisfied (25). Perhaps, this study promotes green environment packaging, thereby making the sustainability feature influencing the consumer buying behavior.

The present consideration fulfills the research gap by profoundly understanding the consumer purchase behavior affected by the product packaging. Packaging does not always have a positive effect on consumer buying intention. Fundamentally, this study highlights the role of packaging and labeling strategies in consumer buying behavior. The packaging and labeling attributes signal the quality and performance of the product, which in turn motivates sustainable consumer behavior. Perhaps, this study incorporates the importance of environmentally friendly practices appealing to health professionals and institutes. In particular, the inherit study aims to examine the relationship between packaging strategies and consumer behavior to pharmaceutical reality. The study takes into thought labeling strategy, influencing the consumer purchase behavior. Indeed, to overcome the research hole, this paper analyzes the mediating role of entrepreneurial innovation in packaging and labeling strategies. It offers the marketers to safeguard the environment through innovative entrepreneurial activities, increasing the environmental performance, thus affecting the consumption pattern of individuals. Besides this, the investigation also discusses the moderating role of (i.e., product traceability knowledge and absorptive capacity) concerning entrepreneurial innovation and consumer behavior.

Significantly, to achieve the goal, the first section sheds light on the importance of the research topic. Further, Section 2 (i.e., literature review) investigates the research questions by presenting various hypotheses in the light of previous developments and researches. In the subsequent section, the research model had applied to examine the study objectives. Along with this, Section 4 presents empirical results while stating the discussion part in Section 5. The last section (i.e., Section 6) concludes the study by discussing the research limitation and encouraging future researchers to derive the work from this study.

## Theoretical Background and Hypothesis Development

The second section illustrates the marketing conceptions from the stakeholder perspective (i.e., producer and consumer), thus discussing the impact of management strategies on consumer buying behavior. This section aims to determine the definitions and theories regarding the research topic. It reviews current market practices and presents relevant material by broadening the scope of the study. The proposed theoretical model elucidates the overview of associations between the variables. In the following section, the academic background had been focused on while elaborating the intended terms: Packaging Strategies, Labeling Strategies, Consumer Purchase Behavior, Entrepreneurial Innovation, Product Traceability Knowledge, and Absorptive Capability. Indeed, all the desired terms had presented in the same sequence in this section.

### Packaging Strategies and Consumer Behavior

Consumers encounter various products in their everyday lives. Products are fundamentally purchased based on the characteristics (e.g., appearance, color, information, packaging, labeling). Out of all product features, packaging plays an integral role in stimulating consumer buying decisions. In the product packaging, visual cues (e.g., color, size, information) assist the consumer in buying decisions. The visual cues help the consumer make the right decision at the point of purchase. It increases the chances of product acceptance and consumption. In the product packaging, the visual indicators positively impact the consumer purchase decision, thus improving the consumer perception of the health products. Indeed, packaging gains consumer attention subsequently, altering consumer perception about the brand.

Product packaging tends to express one's feelings. A change in the product packaging (e.g., logo, color, information) alters the consumer's perception about the brand, making the consumers ignore the brand. Therefore, an effective packaging strategy establishes strong brand communication (26) while enabling consumers to make the right purchasing choice. Likewise, recent research states that organizations significantly adapt packaging strategies, stimulating consumer purchase decisions (23).

The increasing significance of product packaging shows that firms' packaging strategies immensely influence consumer purchase decisions. The packaging developments that are not fully optimized lower the sales, thus adversely affecting consumer buying behavior. Given the statement, the study shows that firms' packaging strategies enhance the buyers' sensibilities in forming a favorable purchase decision (27). The research indicates that many companies have realized the importance of packaging strategies to differentiate the product, thus providing the customers with an optimum purchase experience (2).

Intelligent packaging strategies are a fundamental marketing tool, driving consumers' behavior. Packaging strategies allow the manufacturers to shape the consumers' expectations, subsequently influencing their purchase choices (21). Hence, the literature concludes that packaging strategies work as a differential tool, providing a great value to the manufacturers, thus stimulating consumer buying behavior. Consequently, based on the empirical researches, the hypothesis proposed is as follows.

**H1**: *Packaging strategies positively influence consumer behavior*.

### Labeling Strategies and Consumer Behavior

Brand labeling provides information about the product features. It is a significant element in communicating the information to the end-users (7). It involves a human cognitive process that influences an individual's feelings and actions regarding the product purchase (28). This phenomenon is highly relevant in the case of healthy products as it enables the consumer to identify and evaluate the best options, leading to optimum decision-making.

The product label involves information about the product name, date of expiry, date of manufacture, price, and ingredients. While only judging the external packaging, consumers cannot evaluate the intrinsic attributes (e.g., taste, ingredients, smell, nutrition). In such circumstances, consumers use product labels to judge the quality and performance of the product, ultimately influencing the consumer's purchase intention (29). Labeling information enables the consumer to make healthier choices. The potential information assists the consumer in making essential evaluations while making a purchase decision (30). Similarly, a significant association had recorded between the label strategy and customers' purchase of healthy products (8). Perhaps, the label information (i.e., calorie, ingredients) documented on health products allows the manufactures to record positive consumer reactions (31).

Label information alters the consumers' choices and behaviors, encouraging them to pay for the healthier product (32). The format of the label information motivates the consumer to opt for healthy choices (33). The label characteristics and consumer behavior alter the consumer preferences regarding the product purchase (34). Label disclosure is an important strategic element for consumers (35), extending the consumer interest in the brand (36). Nowadays, manufacturers are recording detailed label information (i.e., ingredients) on the product, thereby assisting the consumer purchasing decisions (37).

In the pharmaceutical market, product composition (i.e., nutrition and ingredients) increases the product credibility. The health labels on the drugs products create a positive image of the brand. In support, this developed brand image symbolizes the perceived healthiness of the product. The consumer purchase intention largely depends on the consumers' understanding of the health claims made on the product packaging. The nutrition information cues make the consumer perceive the product, healthful and reliable. The healthy claims alter the consumer's attitude, influencing the consumer buying choices. This improved healthiness makes the consumer better evaluate the product, thus recoding a positive consumer response (9). Indeed, product labeling strategies provide meaningful information to the consumer while enabling them to make favorable purchasing decisions. Subsequently, the recent finding suggests

**H2**: *Labeling strategies positively influence consumer behavior*.

### Packaging, Labeling Strategies, and Entrepreneurial Innovation

The rapid globalization had significantly expanded the request to improve the transparency level across the supply chain development (38), boosting the stakeholder trust within the brand. This need for effective marketing strategies has intensified the organizations' responsibility to provide product-related knowledge to consumers. This heightened demand for packaging and labeling strategies has facilitated the use of new technologies for entrepreneurial innovation (e.g., product tracking) for facilitating transparent supply chain operations (39).

The entrepreneurial innovation practices ensure the introduction of technological applications (i.e., blockchain technology) to improve the transparency of supply chain processes (40). In support, the study states that EI enhances the packaging strategies while improving transparency (41), thus stimulating customer buying behavior. Consumers encounter various information, which in turn alters their preferences. Buying behaviors largely depend on the health and environmental consequences of the product performance. Given the articulation, the study shows that organic labels on the product packaging stimulate consumer purchase intention (42). Indeed, the details on the label make the consumers change their purchase decision (43). Therefore, the literature concludes

**H3**: *Packaging strategies positively influence entrepreneurial innovation*.
**H4**: *Labeling strategies positively influence entrepreneurial innovation*.

### Entrepreneurial Innovation

New products create value for the consumers (44). As a result, companies are continually innovating, providing value to the end-users in the shape of product features (e.g., packaging, designs, functionality). In particular, entrepreneurial innovation enhances consumer perceptions while encouraging the customers to try the product (45). Product innovation derived from entrepreneurial activities encourages marketers to adopt novel ways to meet the stakeholders' demands. It inspires the firms to adapt to new features satisfying the consumers' needs (46), subsequently influencing customers buying attitudes. In particular, entrepreneurial innovation fulfill the needs of consumers through product innovation. The EI improves the quality of the product while making the consumers buy a high-quality product. Perhaps, the modifications brought by the EI significantly alter the consumer purchasing power by adding valuable product characteristics (47). Consequently, the hypothesis concludes.

**H5**: *Labeling strategies positively influence consumer behavior*.

### Mediating Role of Entrepreneurial Innovation

Innovation in packaging adds value to the product. In this new millennium, the role of entrepreneurial innovation mediated by technological advancement is now gaining considerable attention in production businesses (i.e., the pharmaceutical industry), thus delivering the best quality products to the consumer.

However, global businesses have made the EI practices to enhance product innovation. The EI transforms the production activities, leading the firms to take advantage of the market opportunities (48). The novel innovation (i.e., EI) strengthens customer involvement (49). In support, a study shows that consumer purchase behavior is influenced by the organizations' innovation, thereby leading the firms to achieve competitive advantage (50). Given the statement, research shows that innovation in product packaging boosts the EI potential, thereby improving the business competitiveness (51) and recording proactive consumer behavior.

Over the years, the universe of packaging has gone through numerous innovations and alterations. In the strategic role of the product lifecycle, the packaging strategies influence the consumers' buying behavior. Packaging plays a vital role in developing the consumer perception regarding the product purchaser (52). Consumer perception constantly changes. Therefore, marketers should continually innovate products in various forms (e.g., sizes, color, packaging) to meet consumer demand.

In the healthcare sector, health commodities are the hope for humanity. Consumers seek information before buying the product. Pharmaceutical products positively affect consumers' health (53). Consequently, the demand for health products is immensely increasing. Perhaps, the growing consumer demand requires EI in all aspects of healthcare. Entrepreneurial innovation in pharmaceutical manufacturing has a significant impact on consumers' lives. The pharmaceutical company driven by the EI has influenced the elements of the product development (e.g., packaging, labeling) (54), subsequently increasing the demand for the products. Indeed, the product packaging strategies mediated by entrepreneurial innovation form an essential element in driving the consumer purchase decision.

Furthermore, research states that EI solutions develop new techniques for satisfying consumer needs (55). The EI advances the businesses, fundamentally delivering value to the customers, thus taking advantage of entrepreneurial opportunities (56). The firms' labeling strategies develop a strong foundation of entrepreneurial innovation, thus affecting consumer buying behavior. In this regard, research illustrates that companies are undertaking a high level of innovative practices (57), such as eco-label strategy (58), while transiting the entrepreneurial intentions into consumer positive buying behavior (59). Therefore, based on the prior findings, the following hypothesis is developed.

**H6**: *Packaging strategies and consumer behavior mediated by Entrepreneurial Innovation*.

**H7**: *Labeling strategies and consumer behavior mediated by Entrepreneurial Innovation*.

### Moderating Role of Absorptive Capacity

With the rapid evolution of globalization, the fierce increasing competition has compelled organizations to develop and implement innovative knowledge for business success. Entrepreneurial innovation plays a critical role in exploring potential business opportunities while constantly promoting product innovation. Absorptive capacity illustrates the firm's ability to recognize and value the new knowledge, enabling the innovative information to acquire market opportunities. The knowledge application plays an essential role in achieving organizational goals. The high absorptive capacity tends to speed up the innovation process by minimizing the risk-taking uncertainties (60), thereby encouraging the consumers to make favorable purchasing decisions. Indeed, the dynamic capability enables the companies to implement the EI practices (61), allowing the consumers to make informed decisions.

Moreover, the literature demonstrates that absorptive capacity positively links entrepreneurial innovation capabilities (20), making the organization realize the need for changing consumer behavior (62). Perhaps, absorptive capacity strengthens the dynamics of the information society (63), making the entrepreneurial technological innovation enhance consumer buying behavior. Perhaps, based on the prior studies, the following hypothesis is concluded.

**H8**: *Entrepreneurial innovation and consumer behavior moderated by absorptive capacity*.

### Theory of Planned Behavior

**Figure 1:**
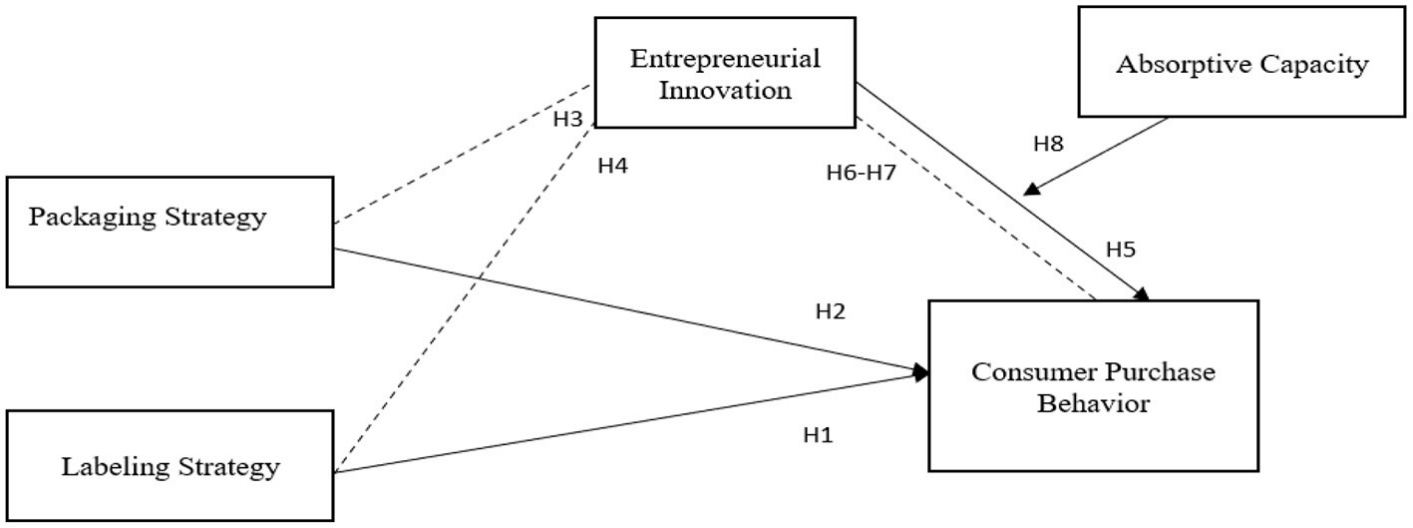
Conceptual framework.

Moreover, numerous theories are being used to describe patients' medicine purchase behavior in literature linked with healthcare, such as reasoned action theory (64) and planned behavior (65, 66). However, they failed to describe consumers' dynamic, evolving and repetitive actions in their purchasing and use of pharmaceutical products in detail. This is exacerbated by the fact that current studies have been performed in various contexts, restricting the observations of the under-investigation phenomena and their functional significance for other populations (67). However, there is a need for a new model for pharmaceuticals to effectively deal with clinicians, regulators, and patients. Following this, the current study is aimed to examine the patient buying behavior in the presence of drug labeling, packaging, and patient awareness. Figure 1 shows study variables.

## Methodology

This study used the quantitative method to measure the packaging and labeling strategies, entrepreneurial innovation, absorptive capacity, and consumer behavior. The target population of this study in Chinese is based on buying health products. Data was collected from consumers of different cities of China; 1,000 questionnaires were distributed to respondents but received 850, 148 questionnaires were not adequately filled. The response rate is 70%. After that, 702 questionnaires were used in the analysis. Convivence sampling method used to collect the data from consumers face to face and electronically. This quantitative data was designed in a questionnaire format. The respondents must answer all questions to assist progress in this quantitative data—adopted questionnaire used as a research instrument to collect data for the research. The study used the 5-point Likert scale to measure the questionnaire's items. The measurement of packaging strategies questionnaire was adopted by (68). The measurement of labeling strategies adopted by (69). In this study, the entrepreneurial innovation measurement scale was adopted by (70). the measurement scale of absorptive capacity adopted by (71). The measurement scale of consumer behavior was adopted by (72, 73).

This study used partial least squares structural equation modeling (PLS-SEM) for analysis purposes. The research model of this study contains first-order constructs. As per statistical literature, the PLS-SEM is suitable software to handle complications of such frameworks (74). The analysis was done through three major processes of Smart PLS: algorithms, bootstrapping and blindfolding (74). The path between constructs was found to be significant.

## Study Results

### Measurement Model

**Figure 2:**
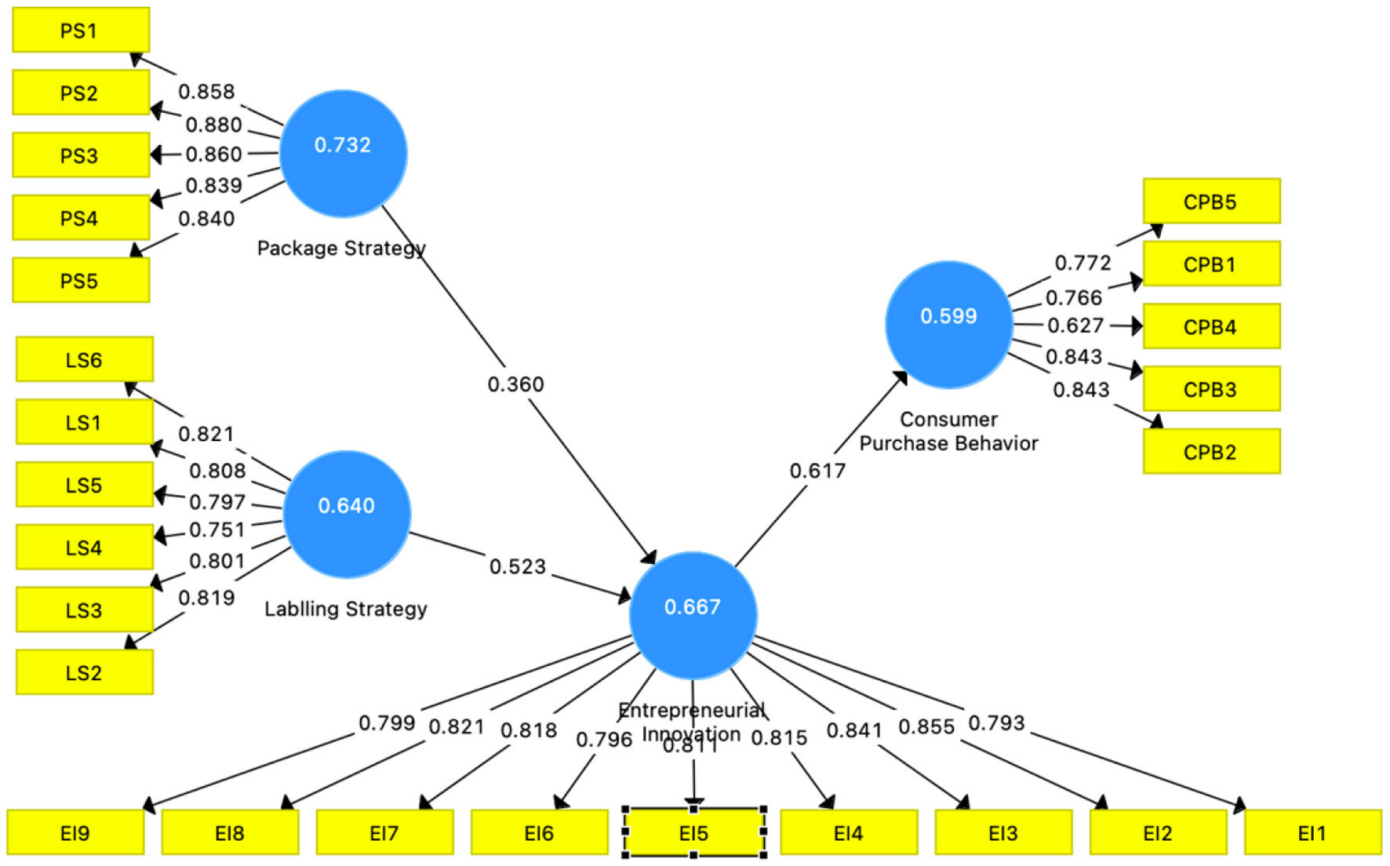
Measurement model.

Consistently, after selecting the software, the next step is a measurement of composite reliability (Figure 2). It is suggested by Hair et al. (74), the reliability value of constructs should be above 0.7, and AVE should be higher than 0.5. In the case of the Variance inflation factor (VIF), the values should be between 1 and 3. The preferable criteria indicate no collinearity issue (75). The values obtained through smart pls algorithms are mentioned in Table 1.

**Table 1:** Factors loading, CR, AVE, and Alpha values.

**Weights**							
	**Items**	**VIF**	**Loading**	**Cronbach's Alpha**	**rho_A**	**Composite reliability**	**Average variance extracted (AVE)**
Consumer purchase behavior				0.839	0.879	0.881	0.599
	CPB1	2.061	0.766				
	CPB2	2.176	0.843				
	CPB3	2.17	0.843				
	CPB4	1.641	0.627				
	CPB5	1.827	0.772				
Entrepreneurial innovation				0.938	0.939	0.947	0.667
	EI1	2.489	0.793				
	EI2	3.254	0.855				
	EI3	3.003	0.841				
	EI4	2.483	0.815				
	EI5	2.416	0.811				
	EI6	2.315	0.796				
	EI7	2.575	0.818				
	EI8	2.873	0.821				
	EI9	2.543	0.799				
Labeling strategy				0.887	0.889	0.914	0.64
	LS1	2.104	0.808				
	LS2	2.223	0.819				
	LS3	2.086	0.801				
	LS4	1.765	0.751				
	LS5	1.949	0.797				
	LS6	2.187	0.821				
Package strategy				0.908	0.909	0.932	0.732
	PS1	2.631	0.858				
	PS2	2.888	0.88				
	PS3	2.532	0.86				
	PS4	2.37	0.839				
	PS5	2.359	0.84				

The discriminant reliability and validity of the model are checked through heterotrait-monotrait ratio (HTMT) and Fornell-Larcker criterion (See Tables 2, 3). The Fornell-Larcker criterion is one of the most used criteria to ensure discriminant validity. It requires the maximum of values at the diagonal level of the table (76). Another criterion of discriminant validity is HTMT measurement. The studies suggest the discriminant values of the study to be maximum at 0.9 and highest values at the diagonal level. This study satisfies the criteria which indicate the successful establishment of the validity of constructs (77).

**Table 2:** Fornell & Larcker.

	**Consumer purchase behavior**	**Entrepreneurial innovation**	**Labeling strategy**	**Package strategy**
Consumer purchase behavior	0.774			
Entrepreneurial innovation	0.617	0.817		
Labeling strategy	0.571	0.719	0.8	
Package strategy	0.631	0.79	0.823	0.855

**Table 3:** HTMT values.

	**Consumer purchase behavior**	**Entrepreneurial innovation**	**Labeling strategy**	**Package strategy**
**Consumer purchase behavior**				
Entrepreneurial innovation	0.643			
Labeling strategy	0.617	0.895		
Package strategy	0.678	0.854	0.915	

### Structural Model

**Figure 3:**
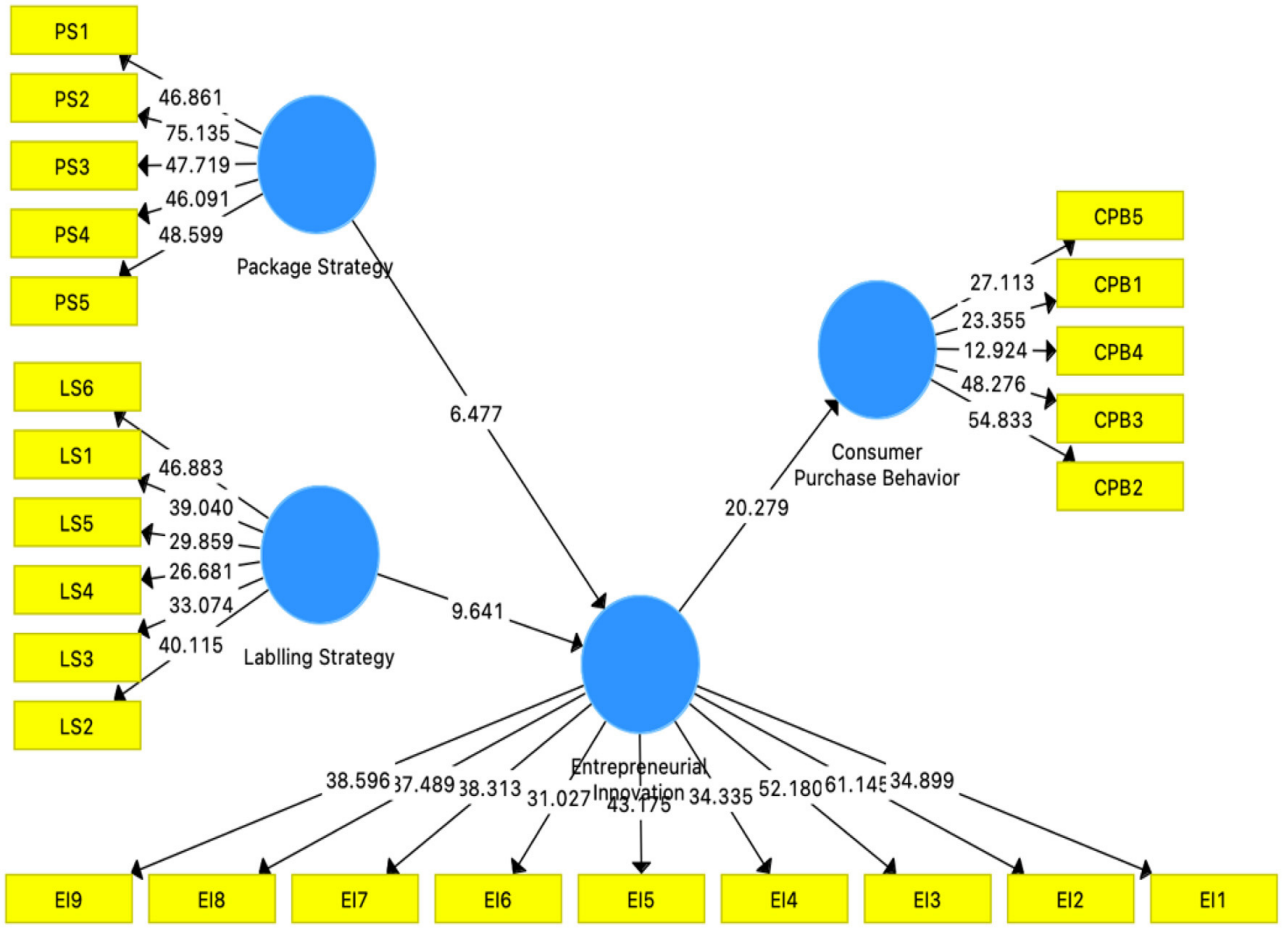
Structural model results.

The hypotheses of this study were analyzed through the bootstrapping procedure, and all relationships were found to be significant, as shown in the table and Figure 3. Furthermore, the study measured the coefficient of determination (R^2^) which measures the predictive powers of the model in a study. It is measured as the correlation of the dependent variable's actual and predictive values (78). The acceptable value of R^2^ ranges from 0 to 1. The values are higher from zero it indicating a higher level of predictive accuracy (79). The value of R^2^ in this study is 0.379, and it meets the required threshold. After that, the study's predictive relevance (Q2) is ensured through blindfolding techniques through PLS-SEM and Stone–Geisser criterion implemented. This technique is used to determine sample predictive powers. The value of Q^2^ should be higher from zero for reflective constructs indicating predictive relevance of dependent variable and path's model (80). The value of Q^2^ of the proposed model is 0.208, meeting the accepted level (See Table 4).

**Table 4:** Hypothesis testing.

**Hypotheses**	**Beta**	**Standard deviation**	***T*-values**	***P*-values**	**F square**	***R* ^2^**	***Q* ^2^**
Entrepreneurial innovation -> consumer purchase behavior	0.617	0.03	20.279	0	0.613	0.379	0.208
Labeling strategy -> entrepreneurial innovation	0.523	0.054	9.641	0	0.306		
Package strategy -> entrepreneurial innovation	0.36	0.056	6.477	0	0.145		
Labeling strategy -> entrepreneurial innovation -> consumer purchase behavior	0.322	0.036	9.067	0			
Package strategy -> entrepreneurial innovation -> consumer purchase behavior	0.222	0.038	5.838	0			
Absorptive capacity*entrepreneurial innovation -> consumer purchase behavior	0.676	0.075	9.077	0			

*p < 0.05.

**Figure 4:**
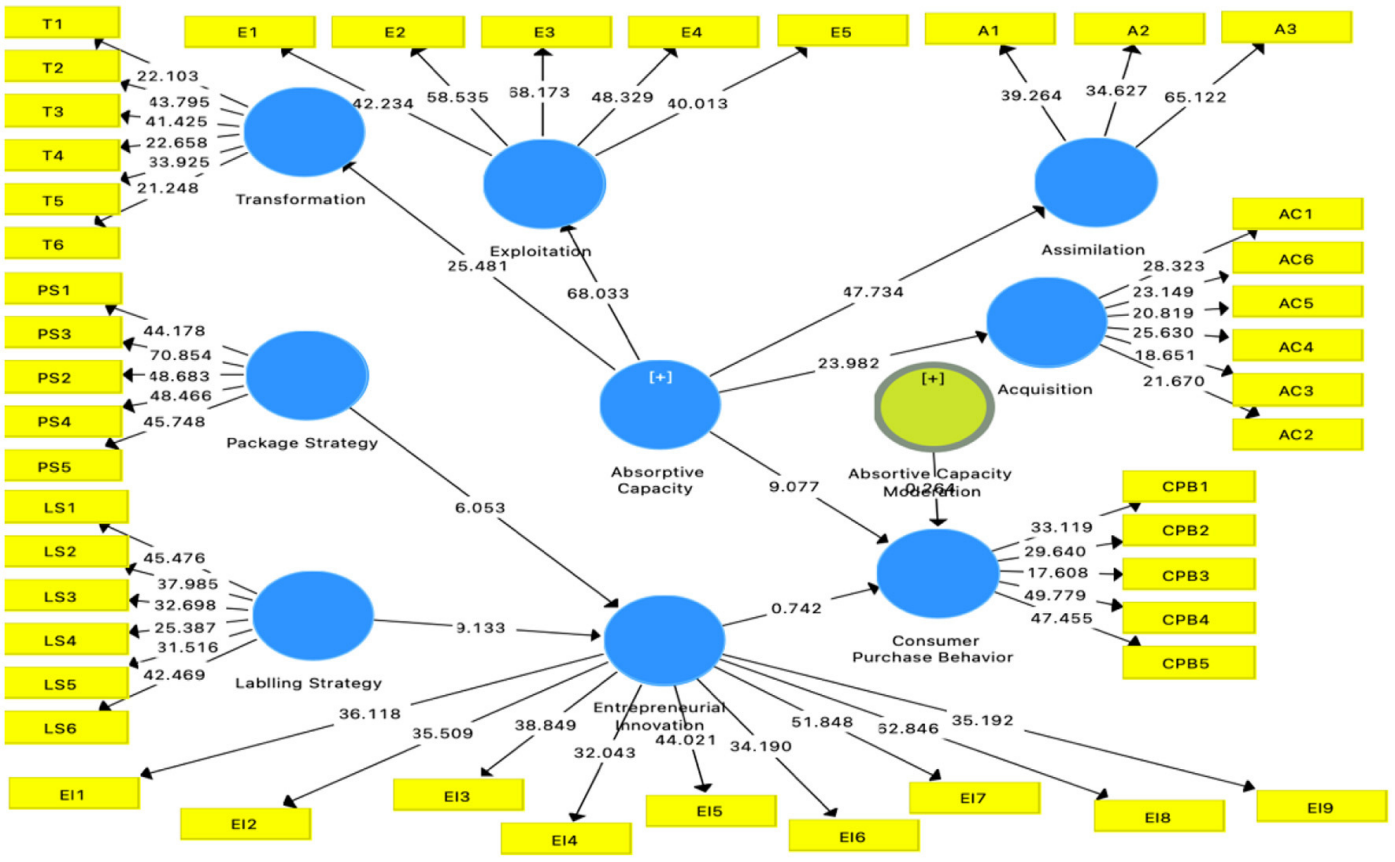
Moderation analysis.

The study proposed the moderation of absorptive capacity. The absorptive capacity has four dimensions: transformation, exploitation, assimilation, and acquisition. The moderation is tested in second-order by using a repetitive indicator approach. The analysis has found that absorptive capacity is significant in the relationship between entrepreneurial innovation and consumer purchase behavior. The moderation was found to be significant at *P* < 0.05, beta value at 0.676, *T*-value 8.9, and *p*-values 0.000 ensured the significance of moderation (See Figure 4).

## Discussion

The previous literature shows that consumers used to make a purchase decision based on the consumer characteristics such as gender, income, ethnicity, and gender. Still, now the time has changed where the consumers have become more concerned about the product knowledge and appearance. Potentially, this section helps understand the study results in the light of prior literature. It discusses the significant role of product packaging, entrepreneurial innovation, and absorptive capacity influencing consumer preferences, choices, and behavior.

Packaging is an influential factor driving consumer behavior. It affects the consumer perception of the brand. Consumer purchase behavior stimulated by various factors includes product quality, price, and packaging. Out of all, the packaging is the unique proposition fostering the consumer purchase intention. In the illustration, the study shows that packaging elements (i.e., color, graphics, size) support the consumer buying decision (81). Accordingly, the study findings show that packaging strategies positively relate to consumers' purchase intention. The results had been found consistent with the previous studies. Packaging portrays the knowledge of good to the consumer, making them buy the product (21).

In healthcare, a rapid shift in consumer need requires marketers' attention. Consumers having health concerns may prefer picking the sealed and unusable products. The healthy labels on health products are a significant predictor of consumer buying behavior. Product packaging develops a psychological association with the consumers. Today, consumers are spending more time assessing the written information on the packaging due to the growing health concerns. Consumers use health-related information and packaging as the fundamental tool for supporting their purchase decision. Perhaps, expanding the consumer awareness through product advancement makes the consumer purchase the product. Given the statement, the study shows that written cues on product labels encourage consumers to make careful decisions (82). Perhaps, this research is consistent with the study findings that records a positive relationship between the labeling strategies and consumer purchase intention.

Moreover, the study shows that entrepreneurial innovation significantly enhances the value of pharmaceutical products (54), subsequently improving consumer purchase choices. Accordingly, our results indicate that entrepreneurial innovation positively mediates the packaging and labeling strategies and consumer purchasing behavior. Indeed, in light of the previous studies, we conclude that entrepreneurial innovation develops a positive relationship between packaging and labeling strategies and consumer buying intention (47). Consistently, the study findings revealed, absorptive capacity positively moderates the relationship between entrepreneurial innovation and consumer purchasing behavior, which is also consistent with the previous literature (20). Indeed, based on the study analysis, all the hypotheses are significantly supported and accepted.

## Conclusion

In today's world of competition, packaging has changed the business environment. In addition to buying high-quality products, consumers are now showing concern toward the product's appearance and information. Significantly, packaging and labeling strategies play a prominent role in enhancing consumer purchase intention. The marketing strategies make marketers design the product with attractive packaging. Packaging makes the product appealing to the consumer (i.e., visual cues), while labeling assists the consumer in making the right purchasing decision. These influential factors satisfy the consumer needs, thus encouraging them to buy the product. Moreover, to strengthen consumer buying, entrepreneurial innovations have captured consumer attention. Due to the rapid growth in fundamental marketing, most pharmaceutical companies are now using entrepreneurial innovations and absorptive capacity as an affirmative tool for driving consumer purchase behavior.

Significantly, this work is presented as transformative consumer research, maximizing consumer knowledge regarding healthcare packaging and labeling strategies. Moreover, it provides the marketers actionable insight into adopting innovative marketing strategies, thus improving consumer buying decisions. This study concluded that packaging and labeling strategies are significantly associated with consumer behavior. It considers the outcomes of consumer purchasing behavior with small firms concerning mixing strategies. Further, packaging strategies and labeling strategies have a significant relation with entrepreneurial innovation; it discovers new ways to gain the competitive edge by introducing new and innovative strategies as the growing perspective of firms. This research shows that entrepreneurial innovation is mediated by packaging, labeling strategies, and consumer behavior. The study's results consider that entrepreneurial innovation is the forerunner of pharmaceutical firms and that entrepreneurial innovation is a mediator in achieving these outcomes. The moderating role of absorptive capacity significantly affects entrepreneurial innovation and consumer purchasing behavior. The implications of the investigated model with innovative marketing strategies give new insights for managerial, policymakers, and research perspectives.

### Study Implications

The study's findings have various theoretical and practical implications. First, this study motivates to add the literature with investigating the packaging strategies, labeling strategies with consumer behavior, and entrepreneurial innovation as a mediator and absorptive capacity as a moderator. This research helps the researcher to examine the important factors in consumer purchasing behavior toward medicines. Second, this research benefits policymakers, owners, and managers in developing countries. Therefore, they must understand firms' packaging strategies and labeling strategies to ease consumers' purchasing. The current study suggests numerous reasons to focus on packaging and labeling strategies, improve the product quality, enhance the image of firms, gain the advantage of competitiveness, conform with environmental pressure, and seek new opportunities. Third, the findings are related to policymakers in developing countries. To inspire and provoke eco-friendlier and consumption and production with packaging and labeling strategies are practical by the policymakers. They can promote better products and services by advising about health's quality information to use.

Indeed, in the light of this current study, a shared value with the consumers, scholars, and practitioners is needed in providing guidelines associated with the organizations' packaging and labeling strategy. This exploration will inspire the stakeholders (i.e., consumer, producer, manufacturer) to design the product strategies, developing a positive association of product information with purchasing behavior. Moreover, the study recommends that manufacturers and producers understand the consumer response toward the marketing strategies by integrating the product design into innovative packaging. Additionally, this study suggests that pharmaceutical firms with inadequate resources will discover more packing and labeling strategies programs. All in all, this study inspires future researchers to formulate the studies for further examining every aspect of packaging, labeling, and product knowledge affecting consumer behavior.

### Limitations and Future Studies

The current study has limitations with cross-sectional research; future research would benefit from longitudinal research design to measure the model over time. Second, the research was conducted on packing and labeling strategies, entrepreneurial innovation, absorptive capacity, and consumer behavior within the specific sector. Future research should be conducted with a new context with a new framework. Thirdly, this study is conducted in a single country (China). Therefore, the researchers should investigate these findings in other countries.

## Data Availability Statement

The raw data supporting the conclusions of this article will be made available by the authors, without undue reservation.

## Ethics Statement

Ethical review and approval was not required for the study on human participants in accordance with the local legislation and institutional requirements. The patients/participants provided their written informed consent to participate in this study.

## Author Contributions

All authors listed have made a substantial, direct, and intellectual contribution to the work and approved it for publication.

## Funding

This study was funded by the Project: Collaborative Innovation Bases for Social Science Application Research of Higher Vocational Colleges in Jiangsu Province-New medical higher vocational education research collaborates innovation bases and Project: The institution system and innovation path of Jiangsu health management modernization in the new era, Project sources: Social Science Foundation in Jiangsu, Project Number: 20XZB018.

## Conflict of Interest

The authors declare that the research was conducted in the absence of any commercial or financial relationships that could be construed as a potential conflict of interest.

## Publisher's Note

All claims expressed in this article are solely those of the authors and do not necessarily represent those of their affiliated organizations, or those of the publisher, the editors and the reviewers. Any product that may be evaluated in this article, or claim that may be made by its manufacturer, is not guaranteed or endorsed by the publisher.
